# Cell-based treatment options facilitate regeneration of cartilage, ligaments and meniscus in demanding conditions of the knee by a whole joint approach

**DOI:** 10.1007/s00167-021-06497-9

**Published:** 2021-03-05

**Authors:** Peter Angele, Denitsa Docheva, Girish Pattappa, Johannes Zellner

**Affiliations:** 1 Sporthopaedicum Regensburg, Hildegard von Bingen Strasse 1, 93053 Regensburg, Germany; 2 Department of Trauma Surgery, University Medical Center of Regensburg, Franz Josef Strauss Allee 11, 93042 Regensburg, Germany; 3 Department of Trauma Surgery, Caritas Hospital St. Josef Regensburg, Landshuter Strasse 65, 93053 Regensburg, Germany

**Keywords:** Autologous chondrocyte transplantation, Knee, Regeneration, Cartilage, Cell-based, Stem cells, Meniscus, Anterior cruciate ligament, Leg axis, Osteotomy

## Abstract

**Purpose:**

This article provides an update on the current therapeutic options for cell-based regenerative treatment of the knee with a critical review of the present literature including a future perspective on the use of regenerative cell-based approaches. Special emphasis has been given on the requirement of a whole joint approach with treatment of comorbidities with aim of knee cartilage restoration, particularly in demanding conditions like early osteoarthritis.

**Methods:**

This narrative review evaluates recent clinical data and published research articles on cell-based regenerative treatment options for cartilage and other structures around the knee

**Results:**

Cell-based regenerative therapies for cartilage repair have become standard practice for the treatment of focal, traumatic chondral defects of the knee. Specifically, matrix-assisted autologous chondrocyte transplantation (MACT) shows satisfactory long-term results regarding radiological, histological and clinical outcome for treatment of large cartilage defects. Data show that regenerative treatment of the knee requires a whole joint approach by addressing all comorbidities including axis deviation, instability or meniscus pathologies. Further development of novel biomaterials and the discovery of alternative cell sources may facilitate the process of cell-based regenerative therapies for all knee structures becoming the gold standard in the future.

**Conclusion:**

Overall, cell-based regenerative cartilage therapy of the knee has shown tremendous development over the last years and has become the standard of care for large and isolated chondral defects. It has shown success in the treatment of traumatic, osteochondral defects but also for degenerative cartilage lesions in the demanding condition of early OA. Future developments and alternative cell sources may help to facilitate cell-based regenerative treatment for all different structures around the knee by a whole joint approach.

**Level of evidence:**

IV.

## Introduction

Regenerative treatment of the knee with restoration of complete knee function following injury is an intriguing therapeutic option, especially in young and active patients. The ultimate goal in these patients is the complete regeneration of the injured tissue for the prevention of osteoarthritis (OA).

Chondral injuries of the knee have a high incidence. Sellards et al. reported that 10–12% of individuals had chondral injuries[95]. Widuchowski et al. evaluated 25,124 knee arthroscopies to quantify the prevalence, location and grade of chondral lesions. They found that 60% of patients had cartilage defects, of which 67% were focal lesions that were mainly located in the retropatellar and medial compartments [105]. In their cohort of more than 30,000 knee arthroscopies, Curl et al. found high-grade cartilage lesions (Outerbridge grades III and IV) in over 60% of their patients [18].The incidence of chondral injuries shows the high societal impact of cartilage defects, as their presence is a risk factor for joint dysfunction that can lead to OA.

This emphasizes the importance of an adequate treatment for cartilage lesions at their formative stages, to prevent the onset and development of OA. Additionally, increasing numbers of younger patients with degenerative cartilage lesions or early OA symptoms after unsuccessful conservative treatment seek therapeutic alternatives to knee arthroplasties, due to risk of revision surgery [9] and only minimal improvements in clinical outcome [37]. Recent developments in biological restoration of injured tissue structures of the knee could fill the treatment gap in such demanding conditions.

Cell-based therapy for treating cartilage defects in the knee joint is routinely performed. Since the introduction of autologous chondrocyte implantation (ACI) by Brittberg et al., large-size cartilage defects can be successfully treated using regenerative medicine approaches [12]. Over the years, increased knowledge has been gained regarding cell source, preparation and surgical techniques. A detailed analysis and correction of all comorbidities like limb malalignment, bony defects, instability and meniscus pathologies are mandatory requirements to achieve a successful outcome of any cartilage restoration procedure[51]. Besides treatment of cartilage defects, cell-based approaches for meniscus and ligament regeneration have been tested in preclinical and, in certain cases, clinical trials.

This article focuses on indications and techniques for regenerative therapy of the knee and highlights developments in cell-based treatment approaches for different structures (e.g., cartilage, ligaments and meniscus) using a whole joint approach. Future directions and limitations for regenerative therapy of the knee will also be discussed.

## Cell-based cartilage repair

### Bone marrow stimulation techniques

**Fig. 1:**
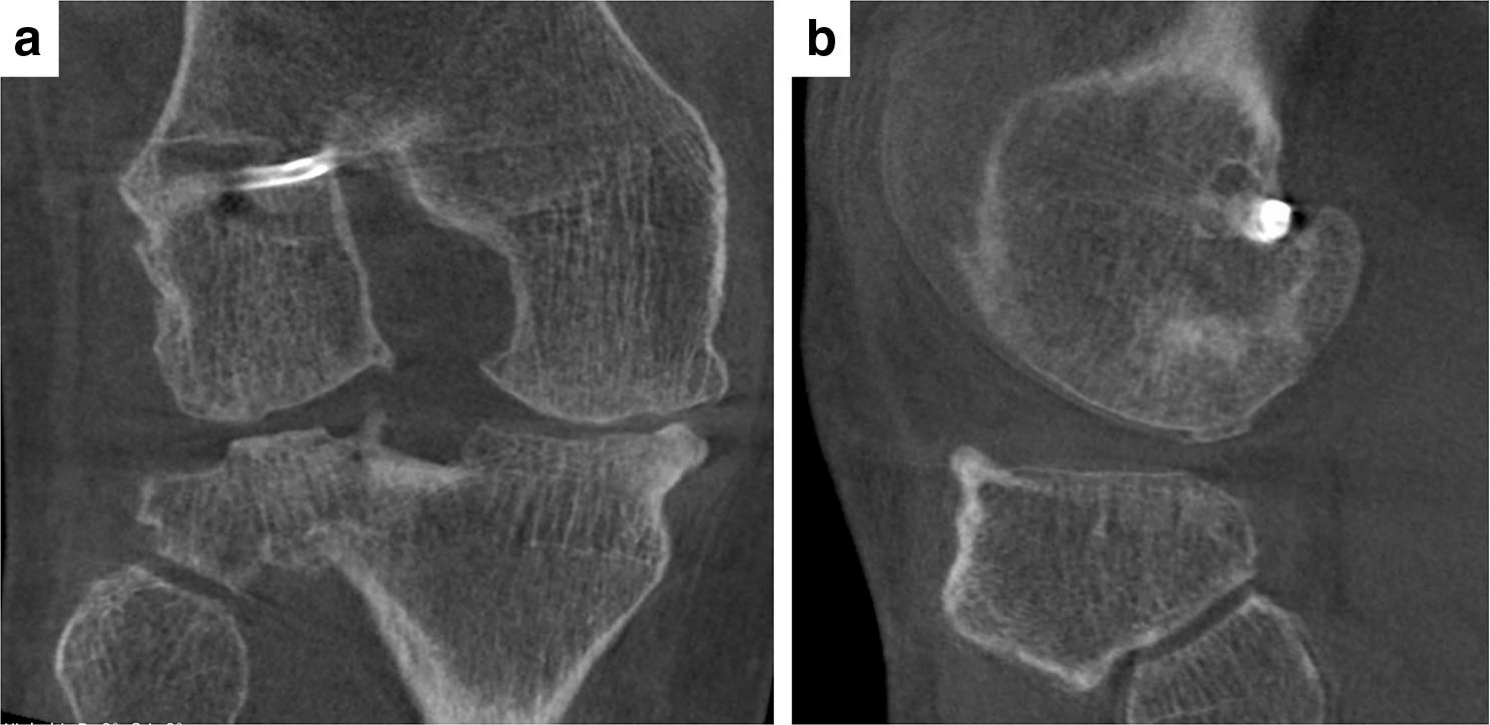
DVT images in an a.p.(A) and lateral(B) view of an ACL-deficient right knee with medial degeneration and intralesional osteophyte at the lateral femoral condyle and the lateral tibia plateau 5 years after microfracture

Amongst reparative options for cartilage treatment, bone marrow stimulation procedures are the most commonly applied technique due to its simplicity and low costs. Its aim is to recruit bone marrow cells from the subchondral bone region, to fill the cartilage defect with cartilage precursor cells. Stem cells migrate from the marrow cavity to the fibrin clot of the chondral defect and lead to the formation of a fibro-cartilaginous tissue with time [59]. In a systematic review, Mithoefer et al. found that microfracturing provides effective functional improvement for at least two years [67]. Steadman et al. who first described this technique, reported satisfactory long-term results [98]. In smaller defects, microfracture shows promising results concerning mobility, reduction of pain and return to sport [46]. However, recent reports demonstrated that after 2–3 years, clinical outcomes following microfracture get increasingly worse, especially in active patients and larger chondral defects. Additionally, the effects of microfracture are age related, as older patients appear not to benefit from this specific treatment [34, 47, 49, 66, 67, 70]. The repair tissue response can be unpredictable. Soft, fibrous and spongiform tissue combined with a degenerative central core is frequently found, and patients need to adjust their activity level in response to knee function [68]. A further reason for the deterioration of the clinical outcome after microfracture with time, may be the development of subchondral sclerosis, cysts or the formation of intralesional osteophytes. Consequently, a complication rate of up to 50% after microfracturing is described in the literature [29]. The results suggest using this procedure only in the treatment of acute and small lesions, and not in large cartilage defects (Fig. 1).

Recently, the technique of microfracturing has been modified to a microdrilling method. The idea of drilling holes through the damaged cartilage area into the subchondral bone marrow space to stimulate repair tissue was first described by Smillie and Pridie [97]. Thermal necrosis was a potential disadvantage that could affect the outcome. The improved modern microdrilling version with arthroscopically applicable narrow-calibre drills up to 4 mm in depth is more reproducible and creates less trauma. Therefore, defect preparation and treatment seem to be more controllable. In an animal model, Chen et al. compared this “micro-Pridie”-drilling method with standard microfracturing via histology. Whilst microfracture caused compacted bone around the created holes that sealed them off from viable bone marrow, drilling cleanly, removed bone from the holes and provided access to channels of the marrow stroma. Heat necrosis was not seen in the drilling group [15]. However, there was no prospective clinical study that showed superior outcome of the microdrilling technique compared to microfracturing [42].

Autologous matrix-induced chondrogenesis (AMIC) uses the concept of cellular recruitment using microfracturing or microdrilling to generate a superclot containing potential repair cells in the cartilage defect. Following bone marrow stimulation, the defect is covered by a cell-free biomaterial. To overcome the shortcomings of the microfracture technique, this enhanced procedure was first described by Behrens et al. [10]. Biomaterials used for this technique include collagen type I, collagen type I/III, hyaluronan or chitosan. The scaffolds can be fixed by sutures or by fibrin glue that enables them to be applied arthroscopically in a one-step procedure. AMIC has demonstrated promising results in terms of functional outcome. In a perspective study, Gille et al. investigated 27 patients up to 62 months post-treatment with a mean defect size of 4.2 cm^2^. According to clinical outcome scores (e.g., ICRS, Tegner, Cincinatti), 87% were satisfied with an increase in functional outcome scores [32]. In a subsequent study, the same authors found a significant decrease in VAS pain score at 1 and 2 years postoperatively [31]. Kusano et al. also detected significant improvements in functional scores and VAS after 29 months but MRI findings showed generally incomplete or inhomogeneous tissue filling [52].

Supporting the chondrogenesis with biomaterials can broaden the indication for this technique to a defect size up to 4 cm [71]. Volz et al. compared the clinical outcome of patients with cartilage lesions of the knee with a mean defect size of 3.6 cm^2^, treated either with microfracturing alone or microfracturing covered with a collagen I/III matrix. After an initial improvement in all groups at 2 years, a significant deterioration in clinical results was seen in the microfracturing group. In contrast, clinical outcome remained stable at five year post-treatment upon biomaterial application [103]. A meta-analysis by Steinwachs et al. demonstrated a significant improvement in scores for VAS, Lysholm score and IKDC at 3 years post-treatment using biomaterial enhanced bone marrow stimulating techniques for a mean defect size of 4.2 cm^2^ [99]. Schagemann et al. observed no relevant difference in mid-term outcome in using either an arthroscopic or mini-open approach for this technique [91].

### Autologous chondrocyte transplantation

Matrix-guided autologous chondrocyte transplantation (MACT) is the standard treatment for large full thickness articular cartilage defects in the knee.

This technique was initially introduced as autologous chondrocyte implantation (ACI) by Brittberg et al. [12]. This is specifically for the treatment of cartilage defects larger than 3 cm^2^ and demonstrated superior long-term success compared to other techniques [11, 84]. The conventional technique is accompanied with periosteum harvest and fixation over the cartilage defect via large skin incisions. Autologous chondrocytes are then injected underneath the periosteal flap. The major drawbacks of conventional autologous chondrocyte transplantation are hypertrophy of the periosteum with high arthroscopy revision rate and up to 20% risk of transplant failure [85].

The MACT was developed to address these problems. Following harvest and a defined culture period, the autologous chondrocytes are seeded on biodegradable scaffolds or as chondrospheres, then implanted into cartilage defects in a second surgery via a mini-open approach or arthroscopically.

Using the new technique of MACT, some of the disadvantages of first-generation ACI, such as transplant hypertrophy, could be eliminated [36, 76, 88]. In a recently published systematic review, a follow-up ranging from 12 to 74 months and a mean defect size of 5.3cm^2^ showed an overall percentage increase in clinical outcome scores of 35.7% and MACT provides success for cartilage repair across various different clinical outcome measures [25]. Mid- to long-term clinical outcome including KOOS, SF-36 and Tegner Score showed favorable results for MACT interestingly with a higher failure rate for treated defects in the femorotibial compartment compared to the patellofemoral lesions [92].

**Fig. 2:**
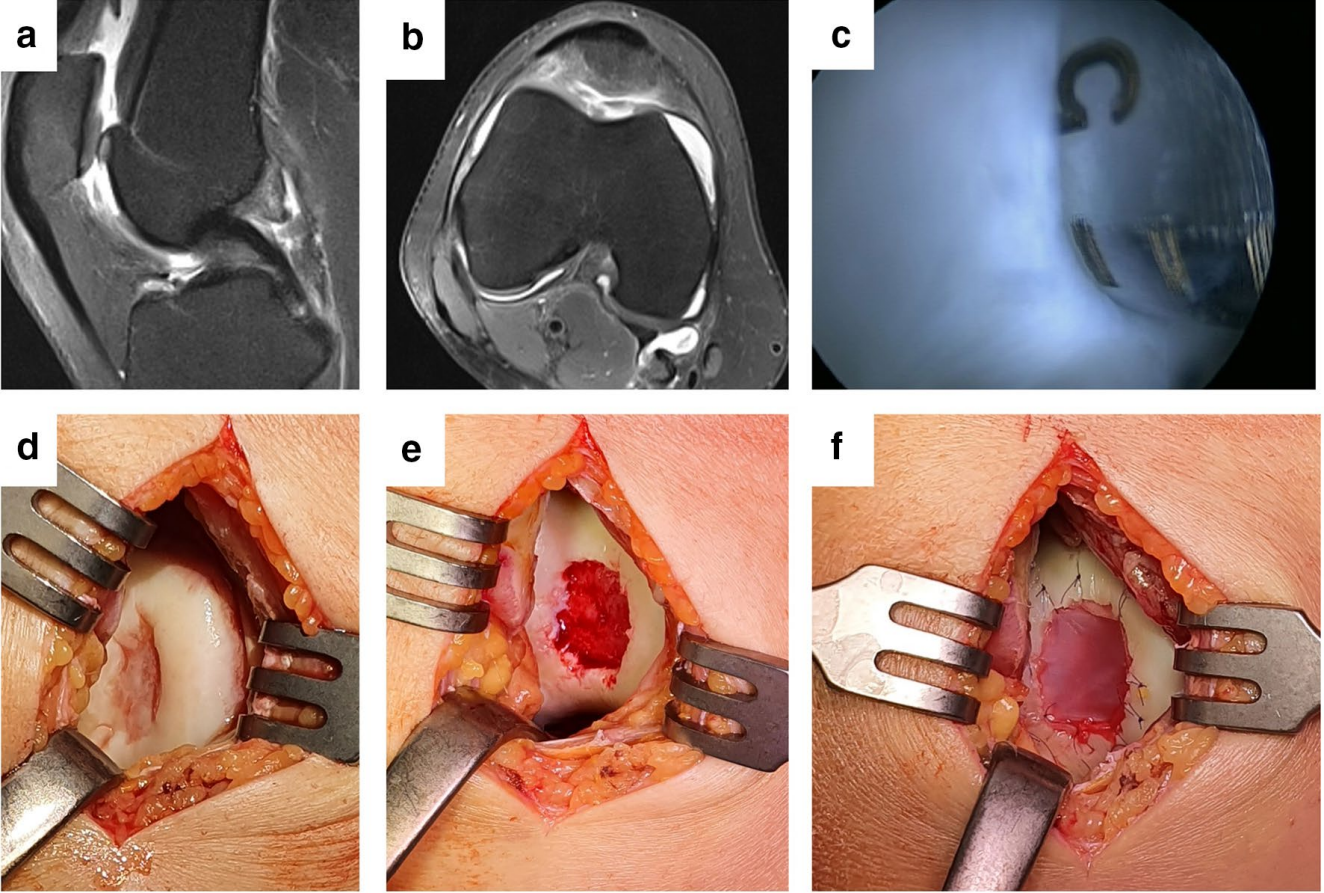
MACT for a large focal degenerative cartilage defect of the trochlea. **a**, **b** Preoperative MRI showing the defect; **c** arthroscopic harvest of an osteochondral plug for chondrocyte culture; **d** cartilage defect in the trochlea; **e** defect preparation; **f** MACT by fixation of the cell-loaded scaffold in the defect with sutures in a mini-open technique

The cellular component (chondrocytes) seems to be relevant for improved outcome. In their review, Kon et al. revealed the advantages of using cells in combination with scaffolds compared to scaffolds alone, for the treatment of cartilage defects, particularly in preclinical studies [48] (Fig. 2).

It has been shown that complete defect filling with functional cartilage tissue correlates with good clinical results. In contrast, incomplete defect filling with undifferentiated scar tissue leads to unsatisfactory scoring results with ongoing pain and poor joint function [38, 43, 53, 68]. In particular, this effect can be observed in larger chondral defects. In a pilot study, we reported that the transplant quality is adequate at the time of surgery for MACT. We retrospectively reviewed 125 patients with large localized cartilage defects (mean defect size: 5 cm^2^) of the knee treated with MACT. Portions of the cell–matrix constructs not implanted into defects were cultured and tested for their potential to form articular cartilage. In vitro assessment of the cell–matrix implants showed chondrogenic differentiation with positive staining for glycosaminoglycans and collagen II, whilst there was an increase in collagen II deposition, as described by ELISA analysis. Clinically, we observed an improvement in median IKDC score from 41 to 67 points at last follow-up. Thus, cartilage extracellular matrix deposition shows adequate implant quality for MACT at the time of implantation and justifies its use for treatment of large cartilage defects [107].

Macroscopic and histological findings play an important role after MACT. In evaluating the quality of the regenerated cartilage tissue, the surface quality and the integration into the surrounding native cartilage are important, alongside defect filling and histological results [102].

A meta-analysis by DiBartola et al. showed a correlation between histological outcome and surgical cartilage repair techniques in the knee, with best results obtained with cell-based treatment strategies [24]. The reason for the superior results after MACT compared to microfracture may be associated with improved defect filling, better cartilage regeneration and a lack of osteophytes in the defect site that can be predominantly seen 4 or 5 years after microfracture [13, 73].

Compared to other reconstructive therapy options for cartilage defects like microfracturing or osteochondral transplantation (OAT), MACT shows the best quality of regenerated tissue [102]. Especially for full thickness cartilage defects larger than 4 cm^2^, the MACT is the recommended therapy in the literature. Other cartilage therapy procedures failed to improve the clinical outcome of large cartilage defects [70]. Bentley et al. demonstrated in a controlled and randomized prospective study that ACI showed significantly better clinical outcome results compared to OAT [11]. In comparison to microfracture, the outcome after MACT for large-size chondral defects (4–10 cm^2^) was significantly better at 2 years post-treatment [8]. Similar long-term results have been described for active patients comparing MACT and microfracture [46]. In another randomized prospective study, Crawford et al. saw significantly more therapy responders in the MACT group compared to the microfracture group after 6, 12 or 24 months. These results correlated with the clinical and functional outcome of patients measured by the KOOS and IKDC Score [17].

Matrix-associated chondrocyte transplantation (MACT) is superior to arthroscopic microfracturing with respect to daily living and sporting activities at 3 years post-treatment [40] with fewer re-operations [75]. In addition, the age-related effects of a cartilage therapy seem to be less significant with the MACT compared to microfracture [74, 86]. Vanlauwe et al. compared MACT with microfracture and showed a significant improvement in patients’ outcome treated with MACT, when the symptoms of the cartilage lesion did not last more than 3 years. On the other hand, in patients with clinical symptoms more than 3 years, MACT failed to significantly improve the functional outcome, compared to microfracture [101]. In order for optimal clinical results to occur, adequate biological repair is required. Consequently, primary cartilage defects should be treated at its earliest possible point to improve the long-term outcome [70, 87]. Due to the need of a two-step surgical procedure with a cell-culture period, MACT results in higher costs. However, despite the high up-front costs, ACI is cost effective over time [50].

In demanding and large osteochondral defects of the knee caused by osteochondritis dissecans or osteonecrosis, MACT provides the chance for a regenerative reconstruction of the joint. Osteochondral treatment with bony defect filling using bone block augmentation from the iliac crest or homologous bone covered with MACT showed promising results with a significant improvement in the IKDC and Cincinnati Score after 2 years. MRI analysis also revealed a good remodeling of the osteochondral unit one year postoperatively [108].

Long-term data describing clinical measures at 10 years post-MACT treatment of chondral defects showed significantly improved clinical and radiological outcome measures in patients with symptomatic and traumatic cartilage lesions [2]. The use of scaffolds in a 3D culture system helps to optimize chondrocyte transplantation both from a biological and a surgical point of view. A prospective follow-up at 15 years showed that arthroscopic MACT offered good and long-lasting results that were stable over time and resulted in a limited number of failures and reinterventions [4]. However, delamination or disturbed fusion to the surrounding native cartilage and subchondral bone remain problems for third-generation ACI. Niethammer et al. reported a revision rate of 23.4% after MACT. The reasons were bone marrow edema, arthrofibrosis and partial graft deficiency. In these cases, arthroscopically performed revision surgery, resulted in significantly improved clinical outcome [77].

Microfracture should solely be used for smal-sized defects and not as a general first-line treatment for cartilage defects, independent of the defect size [64, 82]. Large chondral and osteochondral defects with more than 2.5cm^2^ defect size should be treated with MACT. If microfracture fails as a primary procedure for treatment of a chondral defect, the risk of treatment failure following a second surgery using MACT increases compared to primary ACI [93]. However, there are reports in the literature that demonstrated good results for MACT, even as a re-operation treatment after a previously performed microfracture [106].

### Regenerative treatment with MACT in early osteoarthritis

The best clinical results for MACT can be seen in traumatic chondral lesions and in osteochondrosis dissecans. However, degenerative cartilage defects and chronic lesions are the most frequently seen in clinical practice. Data from the German Cartilage registry showed that 60% of cartilage lesions treated with regenerative therapies were degenerative lesions [72].

Li et al. (2014) found that orthopedic surgeons had problems with the treatment gap for patients with early osteoarthritis of the knee [55]. Especially in patients with degenerative cartilage defects and a long history of pain, a significantly reduced outcome after MACT treatment was observed [28].

For regenerative treatment, it is of utmost importance to differentiate between focal and diffuse early OA. Diffuse early OA cannot be treated with local cell-based implantation, such as chondrocyte transplantation. However, focal early OA is a potential target for MACT [7, 56].

**Fig. 3:**
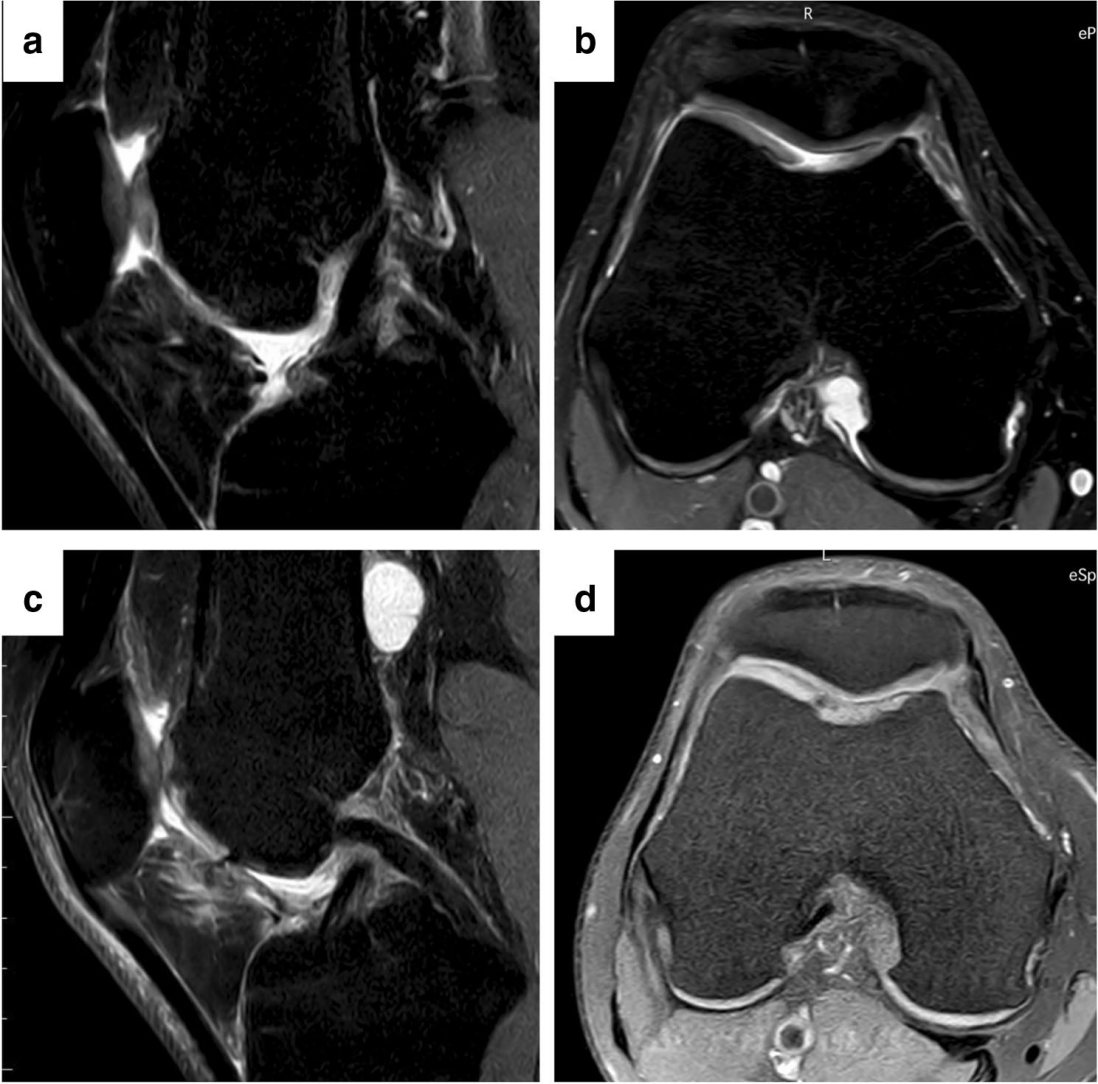
Successful treatment of a grade IV cartilage lesion in the trochlea in early degenerative knee joint environment; **a**, **b** preoperative MRI; **c**, **d** 1 year postoperative MRI with complete cartilage defect filling

A review by Angele et al. described the outcome of regenerative cartilage procedures in patients with focal early OA. Several studies have shown significant improvement of degenerative focal cartilage defects with MACT [7]. In a systematic review, De Windt et al. analyzed 502 patients aged between 36 and 57 who were treated by articular cartilage repair for early OA with ACI performed in 75% of the patients. After a 9-year follow-up, only 2.5–6.5% of the patients had to be converted to an arthroplasty. In particular, ACI shows regenerative potential under early osteoarthritis conditions [20]. Hollander et al. analyzed biopsies of the repair tissue, 16 months after ACI treatment of patients with or without radiological signs of OA. Interestingly, 67% of patient biopsies with OA demonstrated the development of hyaline cartilage, whereas only 36% of patient biopsies without signs of osteoarthritis showed articular cartilage formation [41]. Minas et al. followed 153 patients (mean age: 38.3 years) up to 11 years after treatment with ACI for early OA. Only 8% required conversion to an arthroplasty, whilst 50–75% of the remaining patients improved in WOMAC subscales. ACI treatment in patients with early degenerative changes resulted in pain reduction and an increase in function, so that 92% were able to delay the need for arthroplasty [65]. So MACT may offer improved quality of life for young patients at the onset of OA changes (Fig. 3).

Data from the German Cartilage registry showed that regenerative cartilage treatment resulted in a significantly improved clinical outcome in most of the patients with a low revision rate [83]. Angele et al. saw a significant decrease in joint swelling and pain with an improved knee function after MACT, including those used for the treatment of degenerative cartilage lesions. However, the failure rate after treatment of degenerative cartilage defects was double compared to traumatic defects [5]. Thus, with detailed information of the patient, focal early OA should no longer be considered, as a contraindication for regenerative cell-based cartilage repair procedures.

### Treatment of focal cartilage defects with expanded MSCs

Autologous mesenchymal stem cells (MSCs) are a potential cell source for treatment of large cartilage defects, especially in a degenerative joint. The rationale would be the delivery of fresh cells with minimal influence of degenerative changes and less susceptibility to dedifferentiation compared to articular chondrocytes. In addition, MSCs have a better proliferation rate than chondrocytes and a high potential for chondrogenic differentiation [19].

In a pilot study, Haleem et al. treated five patients with cartilage defects at the femoral condyle with bone marrow-derived MSCs within a platelet rich fibrin glue. After 1 year, all patients’ symptoms improved with positive results in second-look arthroscopies and MRIs showing complete defect filling with surface congruity to native cartilage [35]. Nejadnik et al. analyzed the clinical outcome of patients treated with autologous MSCs compared to patients treated with first-generation ACI for large cartilage defects in the knee. After 2 years, a similar functional outcome regarding IKDC-, Lysholm- or Tegner score was found. The authors concluded that using bone marrow-derived MSCs in cartilage repair is as effective as chondrocytes for articular cartilage repair. In addition, it required one less knee surgery with reduced costs, and minimal donor-site morbidity [69]. In a similar study, Akgun et al. compared matrix-induced autologous MSC implantation versus matrix-induced ACI for treatment of knee chondral defects larger than 2 cm^2^ in 14 patients. At 2-year follow-up, patients treated with bone marrow-derived MSCs showed significantly improved functional outcome and better KOOS subscore results (e.g., pain, symptoms, activities of daily living and sport), than MACT. Control MRIs after 2 years demonstrated that MSC treated cartilage defects with good to excellent defect filling suggesting that these cells can be used to effectively treat full thickness chondral lesions and potentially accelerate recovery [1].

Lineage tracing studies in mice have demonstrated that articular cartilage and synovium have a common developmental origin. Therefore, in vitro studies have discussed the superiority of synovium-derived MSCs for cartilage formation [19, 90]. In a clinical study, Sekiya et al. analyzed the outcome after treatment of focal cartilage lesions of the knee (defect size: 2 cm^2^) with synovial derived MSCs. Following harvest of synovial tissue, the isolated and cultured MSCs were placed into the defect under arthroscopic control after 14 days. The ten treated patients from the initial case series showed an improvement in MRI score, qualitative histology and Lysholm score after 3 years [94].

In conclusion, many authors have demonstrated promising results for regenerative treatment of cartilage lesions of the knee using expanded MSCs. However, at present, in many countries including Europe, regulatory burdens remain a problem for implementing the use of autologous MSCs in daily clinical practice.

### Future perspectives in cell-based regenerative treatment of chondral injuries

**Fig. 4:**
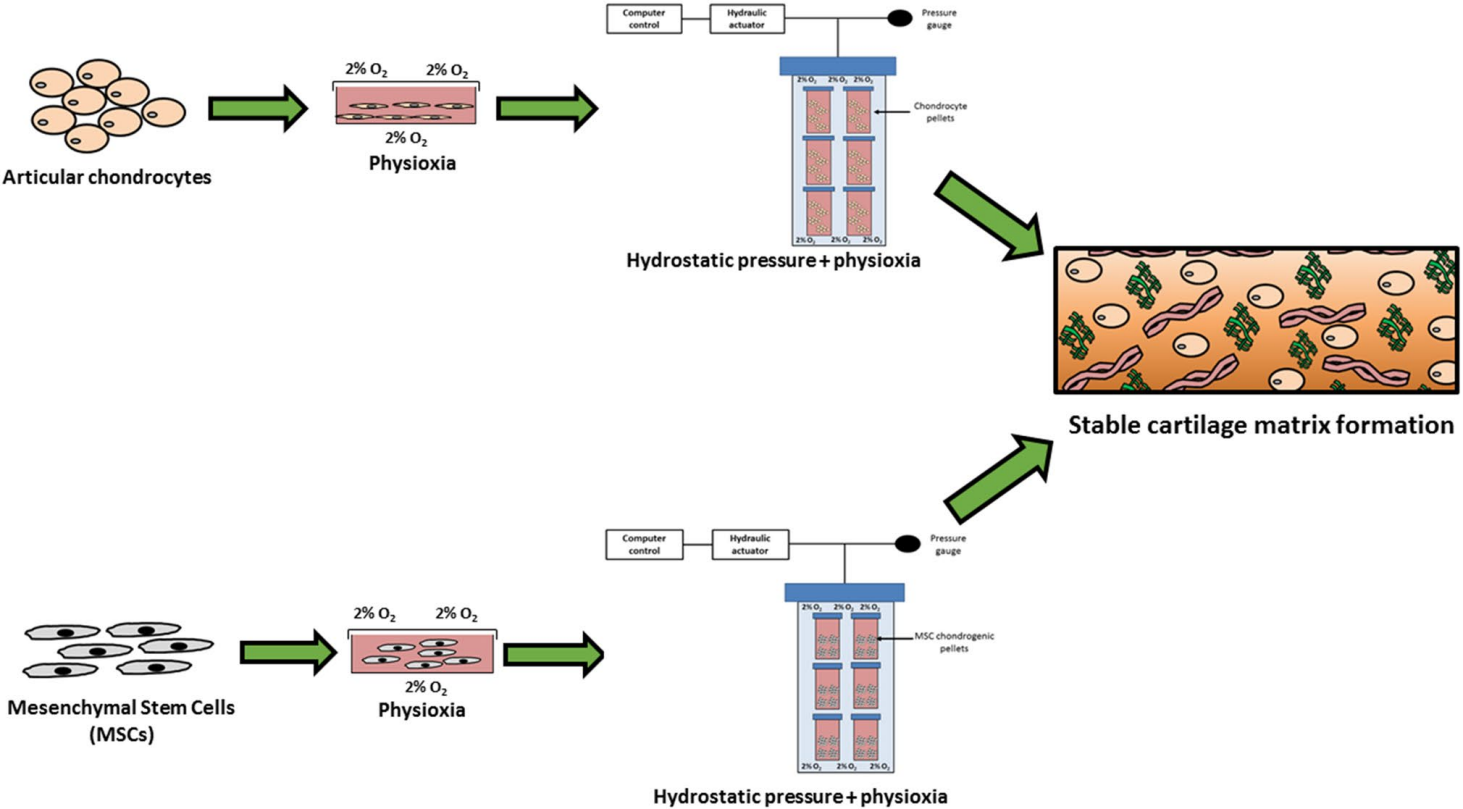
Schematic diagram describing the methods of preconditioning for developing optimal and stable cartilage implants for treating focal early osteoarthritic lesions

Preconditioning of cell-seeded scaffolds prior to implantation into the defect is a potential method for improving cartilage repair procedures. Application of environmental stimuli such as hydrostatic pressure or low oxygen tension during in vitro culture could help promote consistent cell-based techniques. Preclinical data reviewed by Pattappa et al. demonstrated that chondrocytes and MSCs are able to increase matrix formation upon pre-culture under hydrostatic pressure [81]. Crawford et al. described a novel technique of tissue-engineered bovine type I collagen scaffold seeded with autologous chondrocytes preconditioned in a hydrostatic pressure bioreactor prior to implantation [17]. In clinical trials, the authors saw the advantages for treatment of medium- and large-sized chondral lesions after two years with respect to IKDC, KOOS and SF-36 scores. Long-term results are required to evaluate the possible advantage of this process compared to other scaffold-based ACI procedures without biomechanical preconditioning (Fig. 4).

A further parameter for preconditioning cell-based constructs is the application of low oxygen tension or physioxia during the culture period between cell harvest and implantation. In brief, physioxia has been shown to increase matrix production in both chondrocytes and MSCs. In vivo studies utilizing chondrogenic MSCs have demonstrated that there is bone formation upon implantation in a subcutaneous mouse model. The presence of hypertrophic markers is reduced upon culture under physioxia, with subsequent in vivo implantation of physioxia-preconditioned MSCs demonstrating a similar pattern. Furthermore, physioxia-preconditioned cells were able to enhance matrix formation in spite of the presence of inflammatory cytokines that mimic an early OA situation, e.g., interleukin-1 [79, 80]. Combining physioxia and hydrostatic pressure has the potential to improve cell-based therapies for chondral defects in a variety of cartilage etiologies.

Appropriate cell types can also influence the complexity of ACI and simplify surgical procedures. Allogenic chondrocytes can help to reduce donor-site morbidity. In combination with a biocompatible and chondroinductive matrix, allogenic chondrocytes harvested from neonatal donors or from donor knee joints within 24 h of death may be used in a single-stage procedure. Preliminary results demonstrated a safe and effective treatment for cartilage defects with a mean lesion size of 2.7 cm^2^. Clinical outcomes at two years post-op, showed significant improvement over baseline and favorable histological repair tissue [27]. Dhollander et al. reported mid-term results after implantation of alginate beads containing mature, human allogenic chondrocytes in cartilage lesions of the knee. Twenty-one patients were followed up after an average period of 6.3 years and a significant improvement in WOMAC and VAS was observed. However, four failures occurred and MRI evaluation using the MOCART score, only showed moderate scores [22]. Recent studies have discovered the presence of a progenitor cell population within articular cartilage. These cells are known as articular cartilage progenitor cells (ACPs). ACPs are nominally isolated via fibronectin adhesion and shown to be telomerase positive, undergo extensive population doublings, express stem cell CD markers and differentiation to each of the mesodermal lineages (osteogenic, adipogenic and chondrogenic). These cells have been found within both healthy and osteoarthritic cartilage, specifically in the superficial layer of cartilage. An advantage of these cells compared to MSCs is the complete absence of hypertrophic markers during chondrogenesis, especially upon culture under physioxia [3]. In vivo studies demonstrate a good cartilage repair in an animal model [30], although there are no known clinical studies that have used this cell type.

Currently, most ACI procedures have to be performed in a two-step procedure with a period of cell culture in between. Two subsequent operations and consecutively high costs are the disadvantages of these regenerative treatments. Thus, a one-step procedure would be preferable in the future.

A further option for cell-based regenerative cartilage treatment using a one-step procedure is the use of minced cartilage matrix techniques. The cartilage retrieved from marginally degenerative areas or lesion flakes are minced into cartilage fragments (approximately 1 × 1 × 1 mm) and placed back into the defect by fibrin glue or covered with a collagen membrane. A first trial of 27 patients with cartilage defects of 3.1 cm^2^ (average) showed satisfactory clinical outcome regarding pain reduction and improved knee function at two-year follow-up [60]. Longer follow-up and larger cohorts are required to define the benefits of this one-stage procedure. Another technique involves co-culture of MSCs with chondrons (articular chondrocytes with pericellular matrix) from the defect site in a one-step procedure. Recent human trials have described the translation of this technique in thirty-five patients with hyaline cartilage features and good tissue integration observed on second-look arthroscopies. Furthermore, there was found to be an improvement in KOOS score and reduction in pain score (VAS) upon treatment with this technique [21].

### Cell-based treatment of ACL

There is a consensus amongst orthopedic surgeons that knee stability is required for a successful regenerative cartilage treatment. In their review, Mehl et al. showed that chronic instability in ACL-deficient knees is associated with a significant increase in medial meniscal injuries after six months followed by a significant increase in cartilage lesions after 12 months [62]. Similar results were seen by Michalitsis et al., with a significant increase of high-grade cartilage defects in ACL-deficient knees when reconstruction was performed more than 12 months after injury [63]. In their multicenter study, Cox et al. revealed that cartilage lesions and meniscal tears are negative predictors for clinical outcome after ACL reconstruction [16]. Surgeons should take special care to analyze instabilities of the knee prior to regenerative cartilage treatment, as the laxity might precede and predispose ongoing osteoarthritic changes that negatively influences the regenerative milieu.

The question arises whether cell-based regenerative treatment is suitable for ligamentous injuries, such as ACL ruptures. Several biological factors influence the ACL healing process like intraligamentous cytokines and cell repair mechanisms controlled by stem or progenitor cells. MSCs found in the ACL have the potential to differentiate into the ligament linage with tissue specific properties [78]. Prager et al. revealed that the regenerative potential of ACL derived MSCs from old donors was not significantly different in terms of proliferation and differentiation potential compared to that from young donors [89]. However, the clinical efficiency of ACL MSCs for ligament regeneration is unclear, as their availability as a cell source for treatment diminishes with time. Until now, the role of MSCs in ACL regeneration is poorly understood [39].

Clinical use of MSCs for cell-based regenerative treatment of the ACL is limited. In a case series of 29 patients, Centeno et al. showed a subjective improvement in VAS, Lower Extremity Functional Score, IKDC and in MRI appearance of the ACL following injection of autologous BMC in the ACL tear percutaneously [14]. Surgical delivery of bone marrow concentrate was tested to improve bone–tendon healing following ACL reconstruction but showed no differences in MRI evaluation between cell-treated patients and controls [96].

In their systematic review, Di Matteo et al. revealed a paucity of clinical trials investigating the role of stem cells in promoting ACL healing in the case of partial and complete tears. However, other agents of biological augmentation (e.g., PRP) for enhancement of cell-based ACL regeneration might be promising [23]. Koch et al. evaluated the mid-term outcome of a novel healing response technique for partial ACL ruptures that was combined with intraligamentous application of autologous conditioned plasma. At an average follow-up of 33 months, the patients (*n* = 42) showed good to excellent clinical outcome results regarding IKDC, Lysholm, Tegner and Cincinnati Scores. In clinical evaluation, stable Lachman test, negative pivot shift and a significant reduction in anterior–posterior laxity were observed in all patients [45]. In another study that analyzed the enhancement in healing of partial ACL ruptures, Gobbi et al. combined ligament repair with bone marrow stimulation and bone marrow aspirate concentrate. Long-term outcome after a mean duration of 10 years revealed good to excellent clinical results in 73% of the cases with high rates of knee stability restoration and return to preinjury athletic activities [33]. Further studies are needed to translate promising data from basic science and animal studies to daily clinical practice and to extend indications for cell-based regenerative treatment of the ACL to complete ruptures.

### Cell-based treatment of meniscus

The meniscus plays a decisive role in the integrity of the knee joint. This includes shock absorption and transmission, joint stabilization, proprioception, lubrication and nutrition of the articular cartilage [58]. Biomechanical studies have shown that a loss of meniscus integrity leads to changes in kinematics and load distribution in the knee joint. This subsequently increases the pressure on the surrounding native articular cartilage.

The interaction between cartilage and meniscus is manifold and interdependent. Following a meniscus tear, cartilage degeneration usually starts at the corresponding location [57]. Otherwise, meniscal alterations like disorganization of the collagen framework, calcification or decrease of meniscus mechanical resistance, correlates with the location and the degree of cartilage degeneration [26, 54]. Clinical data show that the number of positive responders with respect to clinical outcome for cell-based cartilage treatment decreases with the amount of meniscus loss due to resection at the time of the regenerative therapy procedure (unpublished data from the German Cartilage Registry). According to the increased knowledge concerning the biology and function of the meniscus, there is a consensus to preserve as much meniscus tissue as possible, in the treatment of meniscus injuries, especially in the case of a concomitant cartilage treatment. The vascularization and nutritional situation of the injured meniscus area, as well as the type of meniscus tear, are decisive in the success of a meniscus reconstruction. Therefore, the meniscus still remains a challenging structure for repair and restoration.

To improve the restoration of meniscus tissue, cell-based augmentation for meniscal suture or meniscal replacement is being evaluated. Following an acute meniscal injury, an elevated level of synovium-derived MSCs can be found in the synovial fluid [61]. Due to their potential for differentiation, trophic modulation and good availability, MSCs appear to be the best cell source to support meniscal healing [111]. In different animal models, MSCs showed promising results regarding the development of differentiated meniscus-like repair tissue in small or large meniscus defects or meniscus tears, even in the avascular zone [6, 44, 109, 110]. Vangsness et al. injected allogenic MSCs for meniscal treatment following partial meniscectomy in a clinical setting and detected meniscus regeneration and improvement in knee pain [100]. Whitehouse et al. conducted a first-in-human safety study of five patients with a critical avascular meniscal tear. Autologous MSCs were taken from the iliac crest, expanded, cultured and seeded on a collagen scaffold. These MSC–scaffold constructs were implanted in the meniscal tears and secured in the defect with sutures. At two years post-op, three patients were asymptomatic with functional improvement and no signs of a re-tear in the MRI. Two patients required subsequent meniscectomy due to non-healing after approximately fifteen months [104]. Further clinical studies are needed to show the benefit of cell-based treatment for meniscus injuries. As the meniscus plays an essential role for joint integrity of the knee, its restoration is a key factor in a whole joint approach for cell-based regenerative treatment of the knee.

## Conclusion

Cell-based regenerative cartilage therapy of the knee has shown tremendous development over the last years and has become the standard of care for large and isolated chondral defects. It has shown success in the treatment of traumatic, osteochondral defects but also for degenerative cartilage lesions in the demanding condition of early OA. An improved understanding of the cellular effects of different cell types on cartilage repair, appropriate cell preconditioning techniques, biomechanics, one-step procedures and further developments in cell-based treatment options of other knee joint tissue structures (e.g., ligaments or meniscus), may help to make regenerative therapies become the gold standard in knee restoration.
