# Correction to: Cell-based treatment options facilitate regeneration of cartilage, ligaments and meniscus in demanding conditions of the knee by a whole joint approach

**DOI:** 10.1007/s00167-021-06622-8

**Published:** 2021-06-04

**Authors:** Peter Angele, Denitsa Docheva, Girish Pattappa, Johannes Zellner

**Affiliations:** 1 Sporthopaedicum Regensburg, Hildegard von Bingen Strasse 1, 93053 Regensburg, Germany; 2 Department of Trauma Surgery, University Medical Center of Regensburg, Franz Josef Strauss Allee 11, 93042 Regensburg, Germany; 3 Department of Trauma Surgery, Caritas Hospital St. Josef Regensburg, Landshuter Strasse 65, 93053 Regensburg, Germany

## Correction to: Knee Surgery, Sports Traumatology, Arthroscopy 10.1007/s00167-021-06497-9

The article “Cell‑based treatment options facilitate regeneration of cartilage, ligaments and meniscus in demanding conditions of the knee by a whole joint approach”, written by Peter Angele, Denitsa Docheva, Girish Pattappa and Johannes Zellner was originally published electronically on the publisher’s internet portal on 05 March 2021 without open access. With the author(s)’ decision to opt for Open Choice the copyright of the article changed on 03 June 2021 to © The Author(s) 2021 and the article is forthwith distributed under a Creative Commons Attribution 4.0 International License, which permits use, sharing, adaptation, distribution and reproduction in any medium or format, as long as you give appropriate credit to the original author(s) and the source, provide a link to the Creative Commons licence, and indicate if changes were made. The images or other third party material in this article are included in the article’s Creative Commons licence, unless indicated otherwise in a credit line to the material. If material is not included in the article’s Creative Commons licence and your intended use is not permitted by statutory regulation or exceeds the permitted use, you will need to obtain permission directly from the copyright holder. To view a copy of this licence, visit http://creativecommons.org/licenses/by/4.0/

The original article has been corrected.

